# Extragastrointestinal Stromal Tumor in the Peritoneum: A Case Report

**DOI:** 10.7759/cureus.61411

**Published:** 2024-05-31

**Authors:** Teresa Costa E Silva, Hugo Jorge Alves, Mafalda Vasconcelos, Ana Patrícia Moreira, Bárbara Sousa Picado

**Affiliations:** 1 Internal Medicine, Hospital Beatriz Ângelo, Loures, PRT

**Keywords:** extragastrointestinal stromal tumor, gastrointestinal stromal tumor, peritoneum, immunohistochemistry, dog-1

## Abstract

Gastrointestinal stromal tumors (GIST) are tumors of mesenchymal origin, accounting for less than 1% of the primary neoplasms of the digestive tract, which can affect any segment of the gastrointestinal tract. However, they can also occur in other locations outside the gastrointestinal tract. In such situations, these are known as extragastrointestinal stromal tumors (eGIST).

We present a 58-year-old male, who attended the emergency department due to asthenia, anorexia, heartburn, abdominal pain, and distension, who was ultimately diagnosed with an eGIST in the peritoneum.

The immunohistochemistry pattern of the tumor sample obtained favored this diagnosis, especially demonstrated by the positivity for discovered on GIST protein 1 (DOG1) and negativity of smooth muscle markers.

Due to the rarity of extragastrointestinal tumors and the even greater rarity of those originating in the peritoneum, the authors consider this a pertinent clinical case to be published due to its originality.

## Introduction

Gastrointestinal stromal tumors (GIST) are a group of neoplasms that can affect the entire gastrointestinal tract and account for less than 1% of digestive tract primary neoplasms. They occur predominantly in adults in their fifth decade [1,2]. GIST are mainly found in the stomach, with a prevalence of 60%, followed by the small intestine, with a prevalence of 30% of all cases [3]. The colon, rectum, and esophagus are usually less involved.

GIST were primarily identified in 1983 by their specific immunohistochemical pattern: the absence of smooth muscle markers and consistent expression of CD117 and CD34, resembling that of gastrointestinal pacemaker cells - Cajal interstitial cells, from which these tumors are thought to originate [2].

When arising from other locations besides the gastrointestinal tract, which occurs in 10% of the cases, these tumors are called extragastrointestinal stromal tumors (eGIST). First described in 1999, by Miettinen et al., their origin is still uncertain. Nevertheless, eGIST seem to have identical histological and immunohistochemical characteristics with GIST. They are believed to represent either GIST detached from the gastrointestinal tract wall or independent growths of mesenchymal cells. eGIST may develop in mesentery, omentum, and in very rare cases, in the peritoneum [3].

This uncommon case illustrates the diagnosis of a primary peritoneal eGIST, completely diffused in the patient’s abdomen and pelvis.

## Case presentation

A 58-year-old male, with a previous history of alcohol, tobacco, and drug abuse, pulmonary emphysema, treated hepatitis C virus infection with sustained virologic response, and bipolar disorder, was admitted to the emergency department due to asthenia, anorexia, heartburn, diffuse abdominal pain, and distension with a one-month evolution alongside constipation in the last five days non-responsive to laxatives. He denied any signs of bleeding stools, abdominal trauma, or past surgeries.

Physical examination revealed blood pressure of 121/69 mmHg, heart rate of 96 bpm, and he was apyretic. His abdomen was distended, had flanks’ dullness, and was diffusely painful, especially on the lower quadrants. He also had exuberant edema of lower limbs with a Godet sign.

Laboratory workup showed hemoglobin of 7.7 g/dL, mean corpuscular volume of 99.0 fL, mean corpuscular hemoglobin of 30.0 pg, total bilirubin of 0.3 mg/dL, aspartate aminotransferase of 20.0 UI/L, alanine aminotransferase of 8.0 UI/L, alkaline phosphatase of 91.0 UI/L, gamma-glutamyl transferase of 40.0 UI/L, urea of 28.0mg/dL, creatinine of 0.9 mg/dL, and C-reactive protein 6.2 mg/dL. White blood cells, platelets, international normalized ratio, and albumin were among the reference levels (Table 1).

**Table 1:** Laboratory workup on admission.

Laboratory exams	Laboratory findings	Reference levels
Hemoglobin	7.7 g/dL	13.5-17.5 g/dL
Mean corpuscular volume	99.0 fL	80.0-100.0 fL
Mean corpuscular hemoglobin	30.0 pg	26.0-33.0 pg
White blood cells	7.6x10^9^/L	4.0-10.0x10^9^/L
Platelets	195.0x10^9^/L	150.0-400.0x10^9^/L
International normalized ratio	0.1	0.8-1.2
Aspartate aminotransferase	20.0 UI/L	<40 UI/L
Alanine aminotransferase	8.0 UI/L	<41 UI/L
Alkaline phosphatase	91.0 UI/L	40.0-130.0 UI/L
Gamma-glutamyl transferase	40.0 UI/L	10.0-71.0 UI/L
Total bilirubin	0.3 mg/dL	<1.2 mg/dL
Albumin	3.7 g/dL	3.5-5.2 g/dL
Urea	28.0 mg/dL	13.0-43.0 mg/dL
Creatinine	0.9 mg/dL	0.7-1.2 mg/dL
C-reactive protein	6.2 mg/dL	<0.50 mg/dL

**Figure 1:**
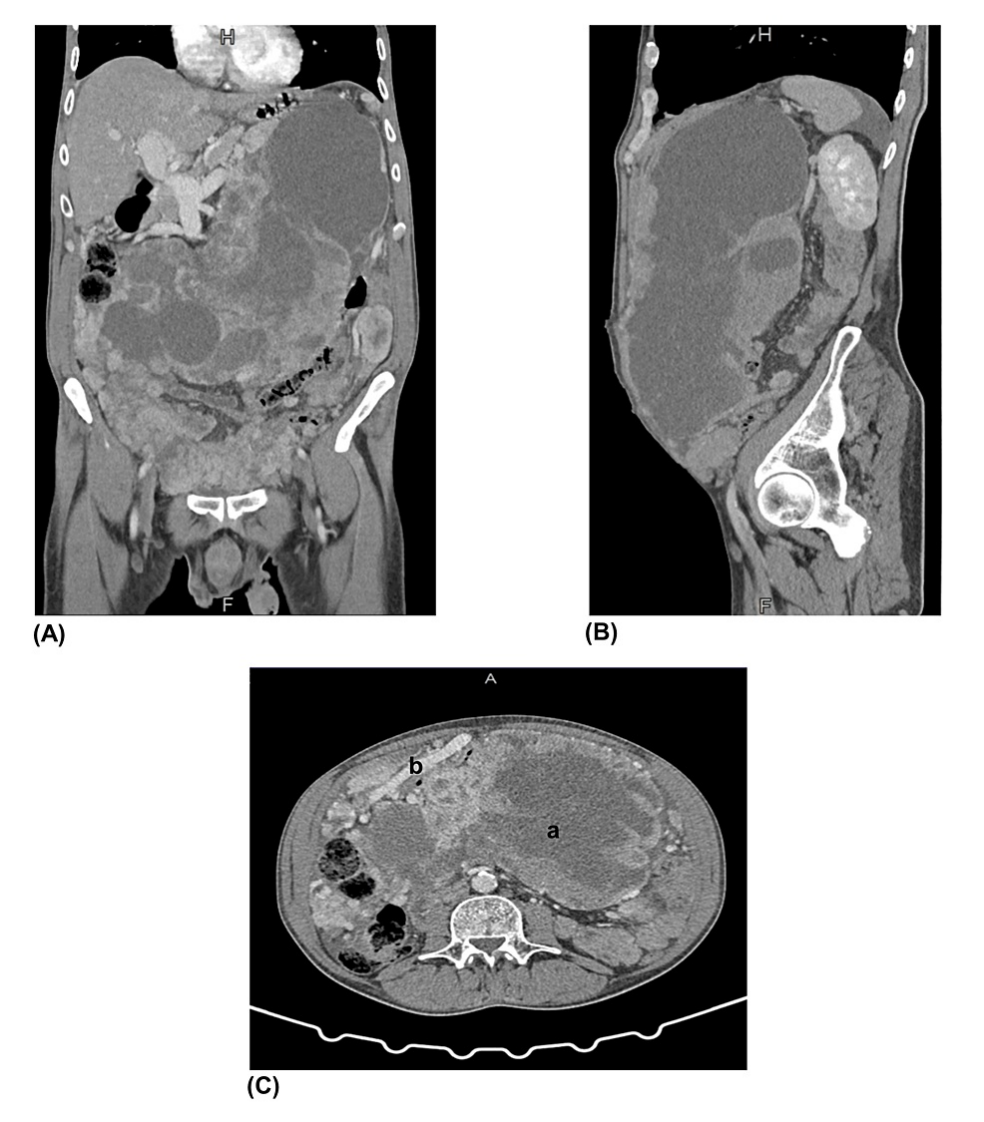
Abdominopelvic contrast-enhanced CT scan. (A) coronal plane, arterial phase; (B) sagittal plane, portal phase; (C) horizontal plane, arterial phase: a - areas of necrotic component with no enhancement; b - dilatation of a vascular structure next to the heterogenous tumor. CT, computed tomography

An abdominopelvic contrast-enhanced computed tomography (CT) scan was conducted demonstrating exuberant filling of the peritoneal cavity by countless solid, heterogeneous lesions, some with a cystic/necrotic component, which exerted a significant mass effect on the intra-abdominal structures, namely mesenteric and vascular (Figure 1). A relatively small amount of ascitic fluid and liver morphology compatible with chronic liver disease with caudate and associated portal vein hypertrophy were also found. No suspicious liver focal lesions were seen.

**Figure 2:**
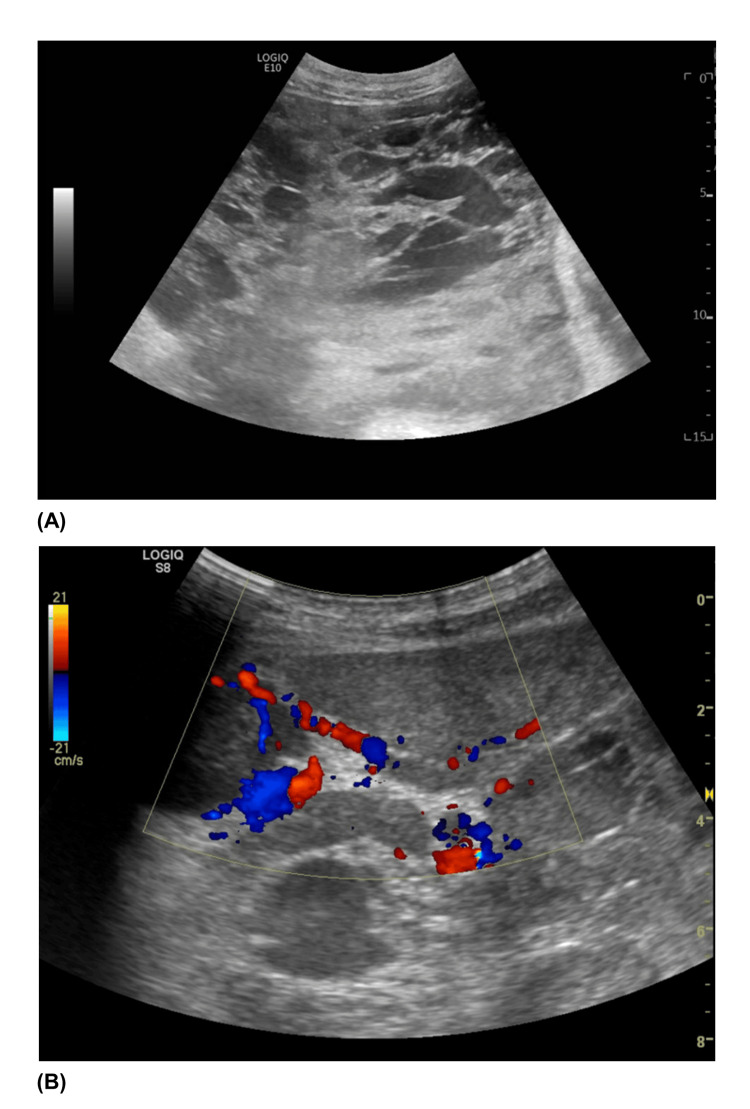
Abdominal ultrasonography. (A) B-mode; (B) Doppler, showing very vascularized nodularity in the peritoneal cavity.

He was then admitted to the ward under conservative treatment and scheduled for further examinations concerning differential diagnosis, namely, neoplastic involvement and non-neoplastic involvement such as sarcoidosis, inflammatory pseudotumor, and encapsulating peritoneal sclerosis. Due to the higher suspicion of neoplastic evolvement of the peritoneum, meriting histologic approach, an ultrasound-guided peritoneum biopsy was done (Figure 2).

**Figure 3:**
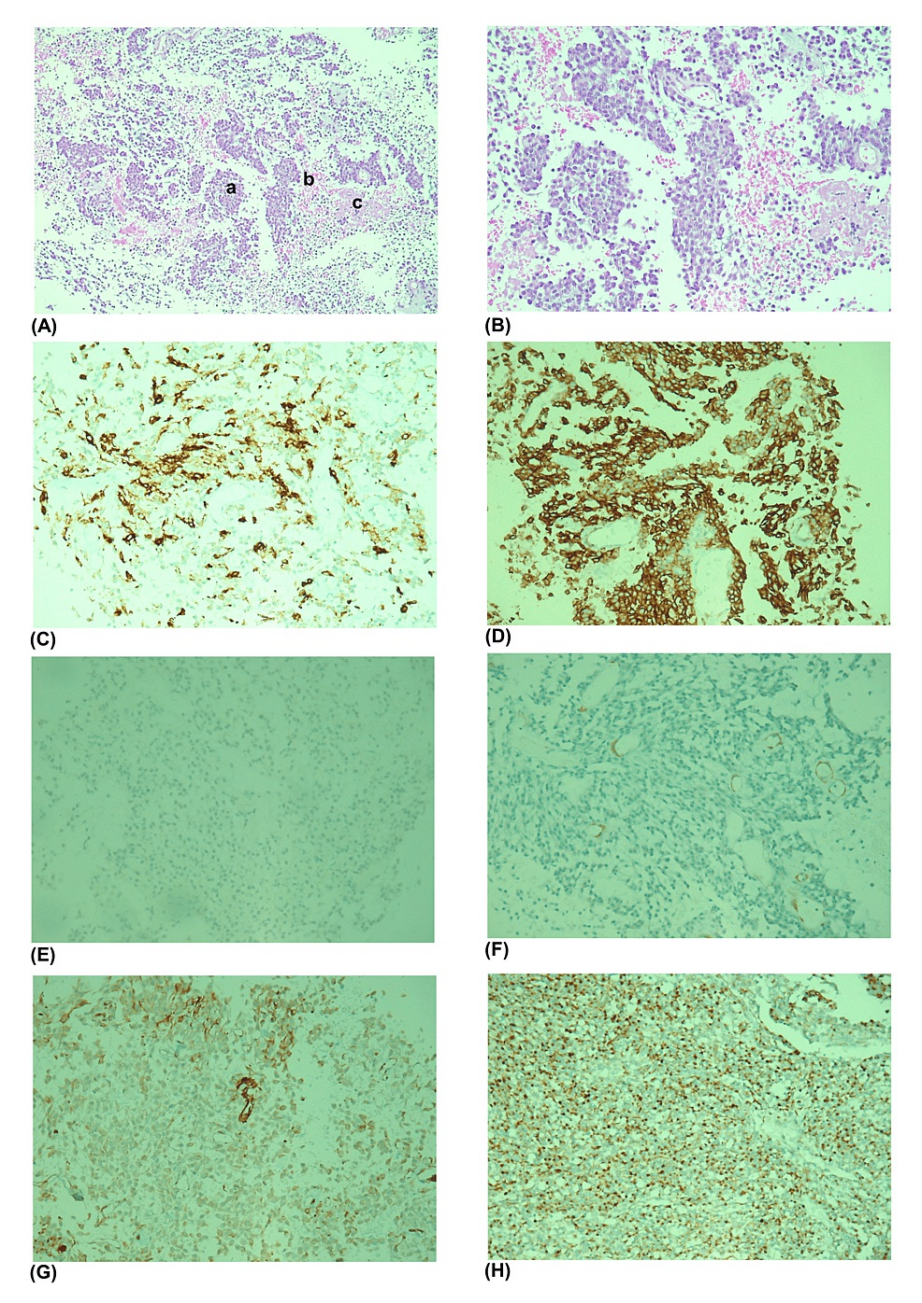
Histological and immunohistochemistry pattern of tumor sample obtained. (A) H&E 10x, a - tumor cells, b - necrosis, and c - peritoneum cells; (B) H&E, 20x; (C) CD117+, multifocal pattern, 20x; (D) DOG1+, 20x; (E) CK AE1/AE3-, 20x; (F) actin-, 20x; (G) WT1-, 20x; and (H) WT1 control, 20x. CK markers helped exclude carcinomatous tumors, actin marker muscular tumors, and WT1 mesothelioma. H&E, hematoxylin and eosin staining; DOG1, discovered on GIST protein 1; CK, cytokeratin; WT1, Wilms’ tumor gene 1; GIST, gastrointestinal stromal tumors

The diminished sample collected held a poorly differentiated neoplasm, with a chordoid pattern, composed of cells with small, monomorphic nuclei, with dense eosinophilic cytoplasm, dispersed in a vacuolated myxochondroid stroma. No mitosis was documented. The immunohistochemical study of the neoplasm revealed cytokeratin (CK) AE1/AE3-, CK8/18-, CK7-, epithelial membrane antigen (EMA)-, protein S100-, tumor protein 63 (p63)-, calponin-, desmin-, actin-, myoglobin-, Wilms’ tumor gene 1 (WT1)-, podoplanin-, CD31-, CD117+ multifocal, and discovered on GIST protein 1 (DOG1)+ (Figure 3).

Concerning the morphological aspects, immunohistochemical profile, and clinical context, the diagnosis of eGIST was established. While waiting for an approach by oncology, he suffered clinical deterioration alongside an infectious process and passed away before staging's conclusion and treatment attempt.

## Discussion

GIST are the most common mesenchymal tumors of the gastrointestinal tract, with an incidence of seven to 14 per one million in the general population [4]. The clinical presentation of eGIST broadly depends on tumor location and size. These tumors grow silently and consequently; they are discovered once they reach a significant size, causing, by that time, symptoms of compression, such as abdominal pain and distension [1]. Our patient suffered from these symptoms as well as constitutional symptoms. Moreover, his heartburn and constipation could also be interpreted as symptoms of compression due to the enormous tumor dimensions. Despite this fact, it was impossible to isolate a proper palpable mass on physical examination because of mass heterogeneity and diffuse characteristics.

The choice to perform a CT scan and omit an abdominal ultrasound on admission was related to constipation and abdominal pain, the last mainly reported on lower quadrants, along with concerning vital signs and laboratory workup not revealing abnormal liver function and enzymology tests. Furthermore, the CT scan and magnetic resonance imaging (MRI) are the standard imaging techniques to characterize these tumors. While small tumors usually appear like a well-defined homogeneous mass, large tumors tend to be ill-defined and heterogeneous, sometimes with calcification and necrosis [5]. In the presented patient, the CT scan showed areas of necrotic component with no enhancement in contrast-enhance images. On the contrary, it can be seen as the significant enhancement of feeding arteries near the tumor, as they are usually extremely vascularized [2].

In cases where the tumor is unresectable, a biopsy will be necessary for diagnostic confirmation and therapeutic decision [2]. Limitations of image-guided biopsy may be related to insufficient sampling of the target mass lesion, as in our case, or by the difficulty in performing a mitotic count on aspiration cytology smears [4].

GIST conception is related to activating mutations in the receptor tyrosine kinases genes KIT, for which CD117 is an extracellular epitope, but also in the platelet-derived growth factor receptor alpha (PDGFRA). The diagnosis of GIST relies heavily on CD117 expression by immunohistochemistry, with eGIST having a similar pattern. However, its demonstration is absent in approximately 4% to 15% of the cases, which can make prompt diagnosis and treatment attempts more difficult with receptor tyrosine kinase inhibitors, namely, imatinib. DOG1 can be used as an immunohistochemistry marker on these tumors with negative expression of CD117 and CD34 [5,6].

In the reported case, the immunohistochemistry pattern of the tumor sample obtained favors the diagnosis of eGIST, especially demonstrated by positivity for DOG1 and negativity of smooth muscle markers, the last excluding differential diagnoses such as mesothelioma and leiomyosarcoma.

These tumors are classified based on their malignant potential into a spectrum of very-low to high-risk tumors [7,8]. Ruiz-Tovar et al. revealed that men, constitutional syndrome, abdominal mass at diagnosis, small bowel and retroperitoneum location, and actin-negative tumors are bad prognostic factors [9]. Even though there is ongoing uncertainty about the most adequate prognostic factors, the tumor size and the mitotic rate are the most consensual among the state of the art published so far [2].

Bearing this in mind, we can presume that this patient had a high-risk tumor and unfavorable prognosis, which proved to be true given the patient's death before any therapeutic option was attempted.

## Conclusions

eGIST are uncommon and diffuse primary eGIST of peritoneum are even rarer. When a CT scan reveals diffuse peritoneal lesions with focal necrosis, eGIST should be considered. Immunohistochemistry also plays an important role in their diagnosis. We believe that the patient’s outcome was mainly determined by the tumor’s size and subsequent symptoms. However, his comorbidities and the following clinical context may also have played a role. Future data is necessary to better define eGIST’s pathogeny, behavior, and treatment.
